# Pharmacognostic Evaluation of *Monarda didyma* L. Growing in Trentino (Northern Italy) for Cosmeceutical Applications

**DOI:** 10.3390/plants13010112

**Published:** 2023-12-30

**Authors:** Antonella Smeriglio, Mariarosaria Ingegneri, Maria Paola Germanò, Luigi Miori, Giulia Battistini, Federica Betuzzi, Paola Malaspina, Domenico Trombetta, Laura Cornara

**Affiliations:** 1 Department of Chemical, Biological, Pharmaceutical and Environmental Sciences, University of Messina, Viale Ferdinando Stagno d’Alcontres 31, 98166 Messina, Italy; antonella.smeriglio@unime.it (A.S.); mariarosaria.ingegneri@unime.it (M.I.); germanom@unime.it (M.P.G.); 2 Areaderma S.r.l., Via per Trento 16, 38042 Baselga di Pinè, Italy; mioriluigi@areaderma.it (L.M.); battistinigiulia@areaderma.it (G.B.); 3 Department of Earth, Environment and Life Sciences, University of Genova, Corso Europa 26, 16132 Genova, Italy; federica.betuzzi@edu.unige.it (F.B.); laura.cornara@unige.it (L.C.)

**Keywords:** Lamiaceae, micro-morphology, anatomy, phytochemistry, polyphenols, anthocyanins, antioxidant, anti-inflammatory, anti-angiogenic, skincare applications

## Abstract

*Monarda didyma* L. (Lamiaceae) is a medicinal and aromatic herb native to eastern North America and now is also cultivated in Northern Italy, which shows terminal heads of bright scarlet-red flowers, subtended by a whorl of red-tinged leafy bracts. Starting from 2018, *M. didyma* flowering tops have been included in the Belfrit List of botanicals. However, to date studies on the crude extract of this plant are still lacking. The aim of the present study was to investigate the morphological and anatomical features of the flowering tops and the phytochemical profile of their ethanolic and hydroglyceric extracts (EE and HGE, respectively). HGE was the richest in total phenols (105.75 ± 5.91 vs. 64.22 ± 3.45 mg/100 mL) and especially in flavonoids (71.60 ± 5.09 vs. 47.70 ± 1.27 mg/100 mL), as confirmed also by LC-DAD-ESI-MS. Fifty-three polyphenols were identified and quantified. Even if they showed a common polyphenolic profile, EE and HGE showed quantitative differences. Flavan-3-ols and anthocyanins were the most expressed metabolites in HGE, whereas flavonols were the most expressed metabolites in EE. These features confer to HGE the highest antioxidant, anti-inflammatory, and anti-angiogenic properties, detected by several in vitro and in vivo assays, highlighting a promising use of this plant extract for skincare applications.

## 1. Introduction

*Monarda didyma* L. is an aromatic herb of the Lamiaceae family, native to eastern North America, and known with different common names including Oswego tea, Bee Balm, and Wild Bergamot. These names are related to different traditional uses: Oswego tea is in reference to the use of the plants leaves for preparing a digestive and expectorant tea by the Oswego Indians of New York State [1]; Bee Balm is related to the use of plant resins to soothe bee stings. The name of Wild Bergamot is instead in reference to the similarity of the flower’s aroma to the bergamot fruit [2]. After the famous Boston Tea Party (1773: colonists protested tea taxes) and tea boycott, *Monarda* leaves were used as a tea substitute also by the European settlers [3]. The toothed, minty aromatic leaves are still used today for their pleasant taste.

In the eighteenth century, the plant was also introduced in Europe, where it began to be cultivated mainly in Austria, Switzerland, and Germany, and then also in Northern Italy, in the Trentino Alto Adige region, thanks to its remarkable ornamental value. The Abbot Angelo Franciosi documented the flora of Veneto in the second volume of his work (1805–1822), encompassing both indigenous plants along the coast and cultivated ornamental species in vegetable gardens. Among these, Franciosi notably mentioned and depicted *M. didyma*, already cultivated in that period in this Italian region [4]. In the middle of the 20th century, *M. didyma* was also introduced in Ukraine, where it has been successfully cultivated as decorative, aromatic, and medicinal plant [5]. Recently, *M. didyma* flowers have been widely cultivated to be used as edible flowers suitable for human consumption [6].

Nowadays, for medicinal purposes, both flowers (“Monardae didyme flores”) and leaves (“Monardae didyme herba”), collected at the time of flowering, are used for the preparation of herbal tea, as well as for refreshing syrups. The plant drug contains anthocyanins (monardine), essential oil, phenols and bitter substances, and tannins. Fresh leaves and flowers, due to their mint-like scent, are also used to flavor salads [7]. Many different therapeutic indications have been reported for this species, including colic, gas, dyspepsia, dysmenorrhea, heart disease, cold, fever, insomnia, and others [8]. The Cherokee Indians were the first to use it in traditional medicine to relieve digestive and respiratory symptoms [9]: for example, preparing a poultice for cold [10]. Starting from 2018, *M. didyma* L. “summitas cum floribus”, corresponding to flowering tops, appears in Annex 1 of the Belfrit List of botanicals allowed in food supplements [11]. Other uses of this plant were reported by Roberts [9], who described the use of the leaves and flowers in decoction to obtain a skin tonic for itchy, dry skin and sunburn.

Different studies are today available on *M. didyma* essential oil [6,7,12,13], which is generally rich in secondary metabolites such as thymol and carvacrol, with antioxidant and anti-inflammatory properties [14]. On the contrary, only few studies concern the micro-morphological features of plant leaves and flowers, as well as the phytochemical and biological properties of their crude extracts [15,16].

Considering this, the aim of the present study was first to investigate the morphological and anatomical features of the flowering tops of *M. didyma* and subsequently to investigate the phytochemical profile and some biological properties, such as the antioxidant, anti-inflammatory, and anti-angiogenic activity, of the raw ethanolic (EE) and hydroglyceric (HGE) extracts obtained from the flowering tops.

## 2. Results

**Figure 1:**
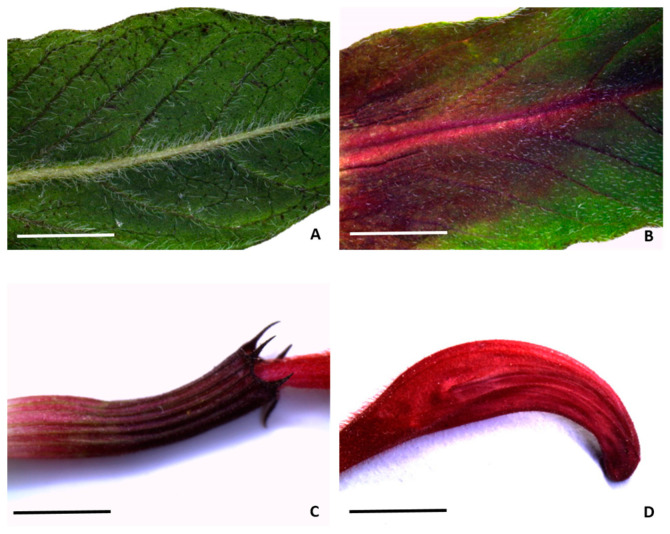
Macro-morphology of the leaf (**A**), bract (**B**), calyx (**C**), and corolla (**D**) of *M. didyma*. Bars = 5 mm.

*M. didyma* flowering tops show dark-green leaves (Figure 1A) clearly distinguishable from the bracts subtending the flower head, characterized by a red-tinged area close to the central rib (Figure 1B). In addition, an intense red pigmentation is also visible throughout the calyx and the corolla (Figure 1C,D, respectively).

**Figure 2:**
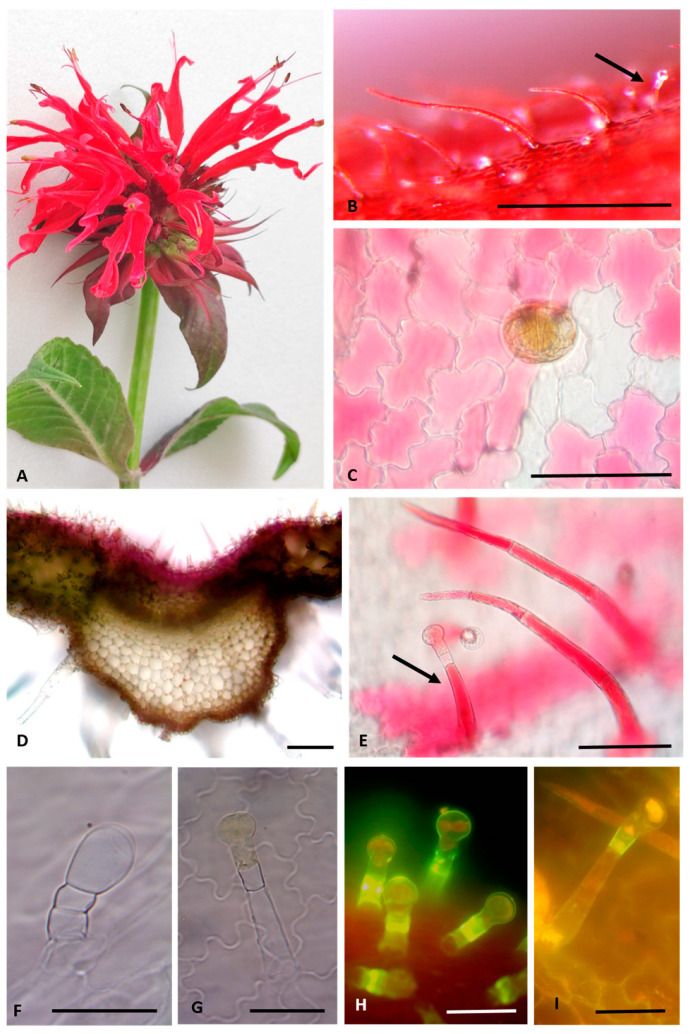
(**A**) Red-scarlet flowers of *M. didyma* L. and red-tinged bracts subtending the flower head. (**B**) Stereomicroscope image showing the corolla surface with long NGTs and CGTs (black arrow). (**C**–**I**) Light microscope images: (**C**) corolla epidermal cells rich in anthocyanins and a peltate glandular trichome; (**D**) transversal section of the bract showing the red-tinged upper epidermis; (**E**) detail of long NGTs and CGTs (black arrow) on the corolla surface; (**F**,**G**) corolla epidermal surface cleared with chloral hydrate highlighting Type II CGT with a unicellular ovoid head (**F**) and Type I CGT with a unicellular spherical head (**G**); (**H**,**I**) corolla surface stained with Fluorol Yellow 088 showing suberin-like substances in the sidewalls of neck cells of Type I short and long CGTs. (**B**) Bar = 0.5 mm; (**C**–**E**) Bars = 100 µm; (**F**–**I**) Bars = 50 µm.

The inflorescence consists of a terminal head of highly compacted cymes, in which the two-lipped, bright scarlet-red flowers mature sequentially from the center outward. The tubular corolla forks into a straight upper lip and a strap-shaped twisted lower lip, with the anthers exerting beyond the upper corolla lobe (Figure 2A).

The characteristic red color of bracts and inflorescences is due to the abundance of anthocyanins, as shown by both stereomicroscopy (Figure 1B–D and Figure 2A) and transmission light microscopy observations (Figure 2B–E). The presence of these red pigments was detected within the epidermal cells of both the corolla (Figure 2B,C) and the bracts’ upper surface (Figure 2D) and even within non-glandular trichomes (Figure 2B,E) and capitate glandular trichomes (Figure 2B,E, arrows). In these latter micrographs, the presence of anthocyanins within the entire non-glandular trichomes is clearly visible, while only the stalk cell of the glandular capitate ones was pigmented. After clearing with chloral hydrate, the trichomes’ morphology became well-visible, but the water-soluble compounds were removed and thus the characteristic red pigmentation disappeared (Figure 2F,G).

**Figure 3:**
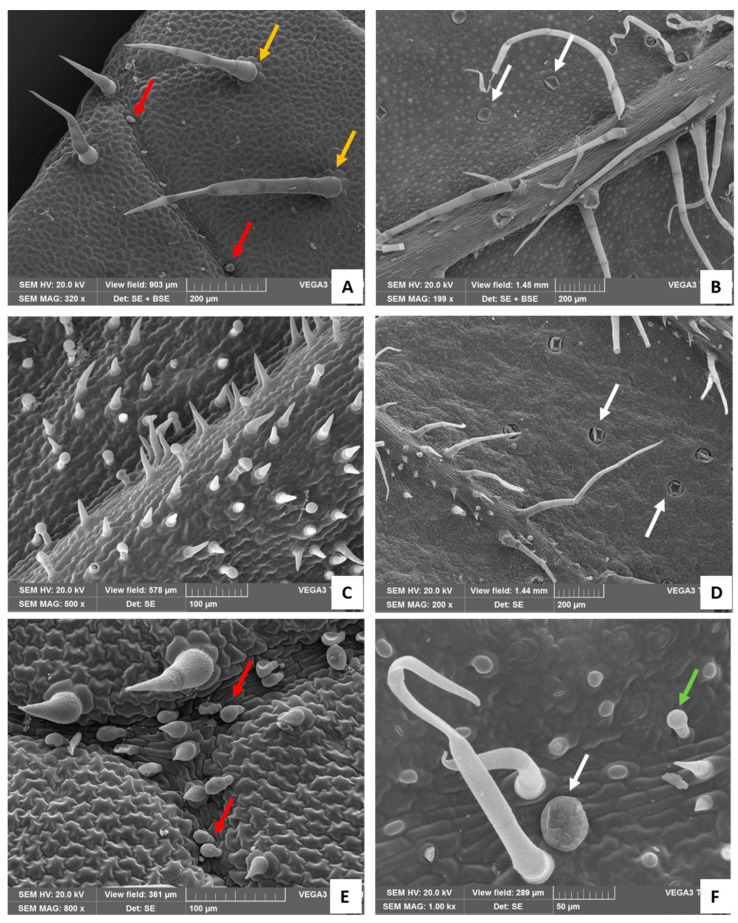
SEM images. (**A**) Adaxial surfaces of the leaf: white arrows show the rosette of epidermal cells around the bases of the long NGTs (orange arrows); red arrows indicate short, stalked CGTs with an oval-shaped head (Type II). (**B**) Abaxial surface of the leaf: white arrows show the peltate glandular trichomes, sunken in the leaf epidermis. (**C**) Adaxial surface of the bract with many stouts and pointed-shaped NGTs and Type I CGTs. (**D**) Abaxial surface of the bracts showing the NGTs on the veins and the peltate glandular trichomes sunken in the epidermis. Detail of the adaxial (**E**) and abaxial (**F**) bracts’ surface showing Type II CGTs (**E**, red arrows), the short Type I CGT (**F**, green arrow), and the peltate glandular trichome (**F**, white arrow).

Two types of non-glandular trichomes (NGTs) were found: (1) long, uniseriate, multicellular (three to eight or more cells), pointed-shaped, and with a slightly striate-to-verrucose surface, which were mainly located on the corolla (Figure 2B,E) and on the leaf (Figure 3A), especially along the veins on the lower surface (Figure 3B); (2) short and stout, two–three celled, pointed-shaped, and with a strongly warty surface, mainly located on the bracts’ upper surface (Figure 2D and Figure 3C,F).

The plant is aromatic due to the mint-like scent of both leaves and flowers related to the presence of many glandular trichomes (GTs). Capitate glandular trichomes (CGTs) could be divided into two types, in relation to the quite variable morphology of the stalk and of the head cell. Type I consisted of a basal cell, a short-to-elongated erected stalk cell (short- or long-stalked CGT), and a neck cell with thickened sidewalls supporting a unicellular spherical head (Figure 2E,G,I). In this neck, sidewall suberin-like substances were present, as shown by Fluorol Yellow staining (Figure 2H,I). Type II corresponded to a short three-celled trichome, with a unicellular ovoid head that ordinarily appeared bent (Figure 2F and Figure 3E, arrows). In addition, many peltate trichomes were distributed in different portions of the plants, mainly in the abaxial surface of leaves and bracts. They consisted of a basal cell, short stalk, and large head rich in lipophilic substances (Figure 2C), containing eight secretory cells arranged in a circle (Figure 3F, white arrow).

### 2.1. Distribution of Non-Glandular and Glandular Trichomes by SEM Analysis

#### 2.1.1. Leaf

NGTs were present on both leaf sides: on the adaxial surface they were medium–long in size and appeared scattered over the entire surface (Figure 3A), while on the abaxial one, they were longer (more than five cells) and mainly concentrated on the midvein (Figure 3B). These trichomes had up to eight epidermal cells arranged in a rosette around the bases (Figure 3A, orange arrows). Short-stalked CGTs with an oval-shaped head (Type II) were present only on the adaxial surfaces mostly near the ribs (Figure 3A, red arrows), while numerous peltate trichomes, sunken in the leaf lamina, were located only on the abaxial surfaces (Figure 3B, white arrows).

#### 2.1.2. Bract

The NGTs were present on both sides of the bract but with a different morphology. Stout and pointed-shaped NGTs, consisting of two–three cells, were uniformly distributed on the adaxial surface and scattered among the Type I short CGTs (Figure 3C). On the abaxial surfaces, both non-glandular stout trichomes and slender non-glandular trichomes, formed by up to five cells, were mainly located on the ribs (Figure 3D). CGTs were present on both adaxial and abaxial surfaces. Two different types of CGTs were detected, namely CGT Type II (Figure 3E, arrows) and CGT Type I (Figure 3F, green arrow). Additionally, several large peltate trichomes were also present, generally sunken in the abaxial epidermis (Figure 3D,F, white arrows).

#### 2.1.3. Calyx and Corolla

**Figure 4:**
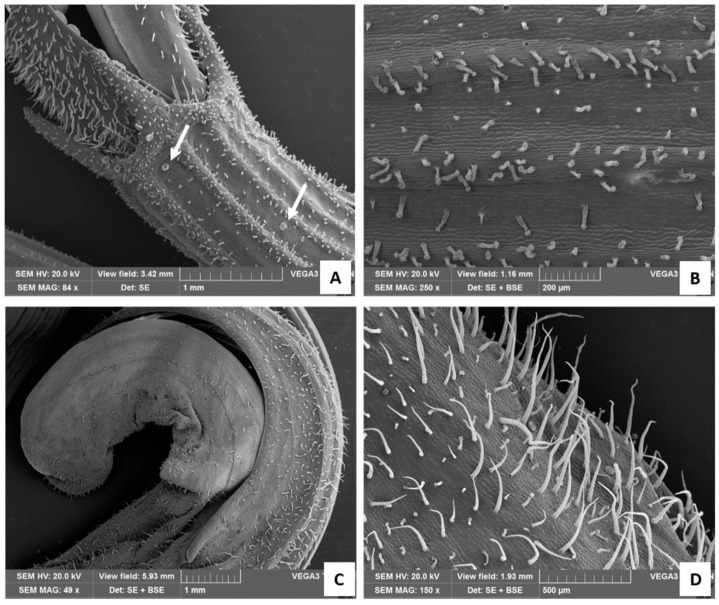
SEM images of the calix (**A**,**B**) and of the corolla (**C**,**D**). (**A**) Detail of the calyx teeth surmounting the corolla, with white arrows showing peltate glandular trichomes; (**B**) calix at higher magnification; (**C**) apical portion of the tubular corolla; (**D**) corolla at higher magnification showing many long and slender NGTs, Type I long-stalked CGTs, and few peltate trichomes.

The calyx showed both capitate and peltate glandular trichomes. Long-stalked CGTs were mainly present on the ribs (Figure 4A,B), while rare peltate ones were located between the ribs (Figure 4A, arrows). The calyx teeth surmounting the corolla were densely covered with trichomes, mostly represented by both peltate and CGTs and to a lesser extent by stout, short NGTs (Figure 4A). The corolla was characterized by the presence of many long and slender NGTs, as well as by Type I long-stalked CGTs and few peltate trichomes (Figure 4C,D).

### 2.2. Phytochemical Analyses

The phytochemical profile of EE and HGE was first investigated by spectrophotometric methods aimed at quantifying the total phenols, total flavonoids, and some sub-classes such as anthocyanins and flavan-3-ols. The latter ones together with proanthocyanidins also allows us to establish the polymerization degree of the extracts under examination. The polymerization index is in fact given by the ratio between the content of flavan-3-ols, determined through the vanillic index test, and the content of proanthocyanidins. Since the proanthocyanidins are oligomers of flavan-3-ols, specifically catechin and epicatechin and their gallic acid esters, if this ratio is much higher than one, it indicates an abundance of monomeric molecules. These first results are shown in Table 1.

HGE proved to be the richest extract not only in terms of total phenols, but also in terms of total flavonoids, anthocyanins, flavan-3-ols, and proanthocyanidins, with statistically significant differences (*p* < 0.001) with respect to EE. Furthermore, this extract also showed the highest polymerization index (*p* < 0.001), highlighting a polyphenols profile very rich in monomeric molecules.

**Figure 5:**
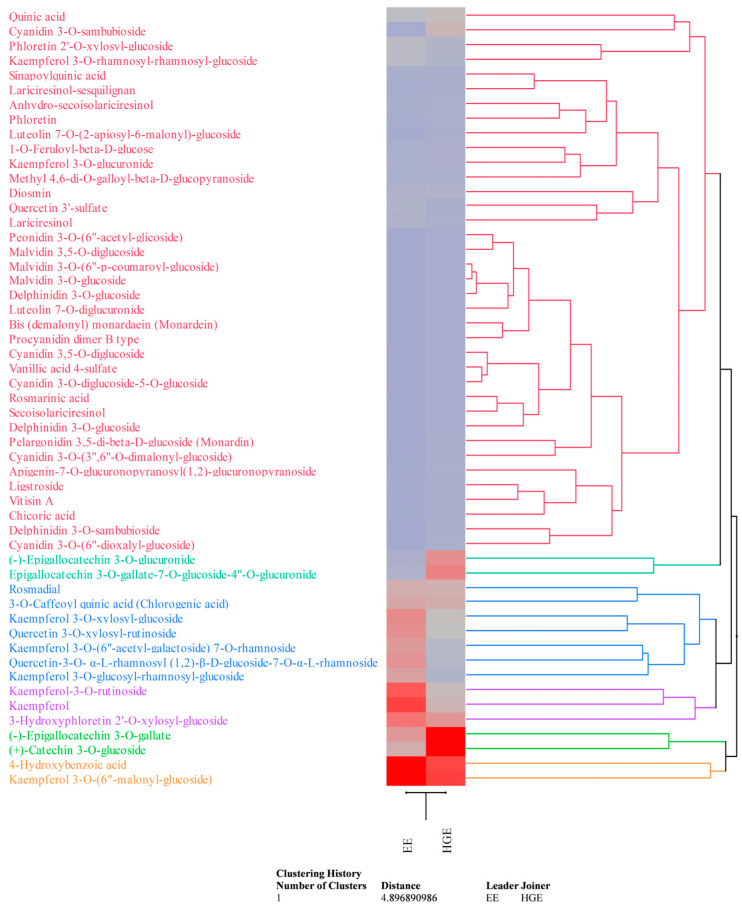
Agglomerative hierarchical clustering analysis of the phytochemical data of *M. didyma* flowering tops EE and HGE obtained by LC-DAD-ESI-MS analysis. The heatmap shows the expression pattern of the identified metabolites, indicating in red and blue the most and the least expressed metabolites, respectively. Color density indicates the fold change between the investigated extracts.

These preliminary data were confirmed by the LC-DAD-ESI-MS analysis (Table 2). Compounds were detected and tentatively identified by comparison of mass and UV–Vis’s spectra with literature data and online free consulting spectra databases, as well as with commercially available standards (see Table 2 footnotes for details). Quantification was carried out by building external calibration curves with commercially available HPLC reference standards (purity ≥ 95%) purchased from Merck (Darmstadt, Germany) and Extrasynthese (Geney, France) when available, or using external calibration curves of structural analogues (see Table 2 footnotes for details). Data, which are the mean ± standard deviation of three independent analyses in triplicate (*n* = 3), were expressed as mg/100 mL of LE. HGE appears, according to previous results, the richest in secondary metabolites (83.33 mg/100 mL vs. 54.92 mg/100 mL of EE), particularly in flavonoids (67.29 mg/100 mL vs. 42.20 mg/100 mL of EE). Although the two extracts show a common phytochemical profile, the expression of each metabolite, in quantitative terms, often varies in a statistically significant manner (*p* < 0.05, Table 2), as it possible to observe also by the agglomerative hierarchical clustering analysis (HCA, Figure 5). Indeed, the heatmap shows the expression pattern of the identified metabolites, indicating the most and least expressed metabolites in dark red and blue, respectively. The color density indicates the fold change between the investigated extracts, allowing us to easily and immediately observe how the expression of each metabolite changes depending on the extraction solvent used.

Flavonols are the most abundant flavonoids class in EE (32.28 mg/100 mL), followed by flavan-3-ols (4.59 mg/100 mL), anthocyanins (0.32 mg/100 mL), and others (5.02 mg/100 mL). On the contrary, HGE showed a higher content of flavan-3-ols (39.56 mg/100 mL), followed by flavonols (19.13 mg/100 mL), anthocyanins (3.01 mg/100 mL), and others (5.59 mg/100 mL). Both extracts showed a discreet amount of phenolic acids and their glycosylated and esterified derivatives (10.32 mg/100 mL vs. 13.08 mg/100 mL for EE and HGE, respectively); on the contrary, only a modest content of lignans (0.70 mg/100 mL vs. 0.38 mg/100 mL for EE and HGE, respectively) and other minor compounds (1.69 mg/100 mL vs. 2.55 mg/100 mL for EE and HGE, respectively) was detected. Kaempferol and quercetin derivatives are the most abundant flavonols detected in both extracts, whereas epigallocathechins the most abundant flavan-3-ols (Table 2, Figure 5). Kaempferol 3-*O*-(6”-malonyl-glucoside) was the most abundant compound in EE (Table 2, Figure 5 orange cluster), followed by 4-hydroxybenzoic acid (Figure 5, orange cluster), kaempferol, kaempferol-3-*O*-rutinoside, and 3-hydroxyphloretin 2′-*O*-xylosyl-glucoside (Figure 5, violet cluster). Conversely, (+)-Catechin 3-*O*-glucoside was the most abundant compound in HGE (Table 2, Figure 5 light-green cluster), followed by (-)-epigallocatechin 3-*O*-gallate (Figure 5, light-green cluster), kaempferol 3-*O*-(6″-malonyl-glucoside) and 4-hydroxybenzoic acid (Figure 5, orange cluster), and epigallocatechin 3-*O*-gallate-7-*O*-glucoside-4″-*O*-glucuronide (Figure 5, dark-green cluster). The HCA essentially demonstrates that the extractive solvents used, also given the similar polarity, did not affect the qualitative phytochemical profile of the extracts examined as much as the quantitative profile (Figure 5). Furthermore, as can be seen from the dendrogram and the related heatmap, the compounds belonging to the red cluster are those which are least affected by the different extraction capacity of the solvents used, although the anthocyanins, and particularly the cyanidin-3-*O*-sambubioside, show a greater affinity for the hydroglyceric mixture.

### 2.3. Biological Properties

The free radical scavenging activity of EE and HGE against 2,4,6-tris(2-pyridyl)-s-triazine (tptz), 2,2-diphenyl-1-picrylhydrazyl (DPPH), 2,2′-azino-bis(3-ethylbenzothiazoline-6-sulfonic acid) (ABTS), and 2,2′-azobis(2-methylpropionamidine) dihydrochloride (AAPH) was investigated by several in vitro spectrophotometric and spectrofluorimetric assays (FRAP, DPPT, TEAC, and ORAC, respectively) based on different mechanisms and reaction environments. On the contrary, the anti-inflammatory activity was investigated by two different assays: the heat-induced bovine serum albumin denaturation assay (ADA), and an enzymatic assay which evaluates the ability of extracts to inhibit the protease activity (PIA). Results, which are expressed as the half-inhibitory concentration (IC_50_) with the respective confidence limits (C.L.) at 95%, are shown in Table 3.

**Figure 6:**
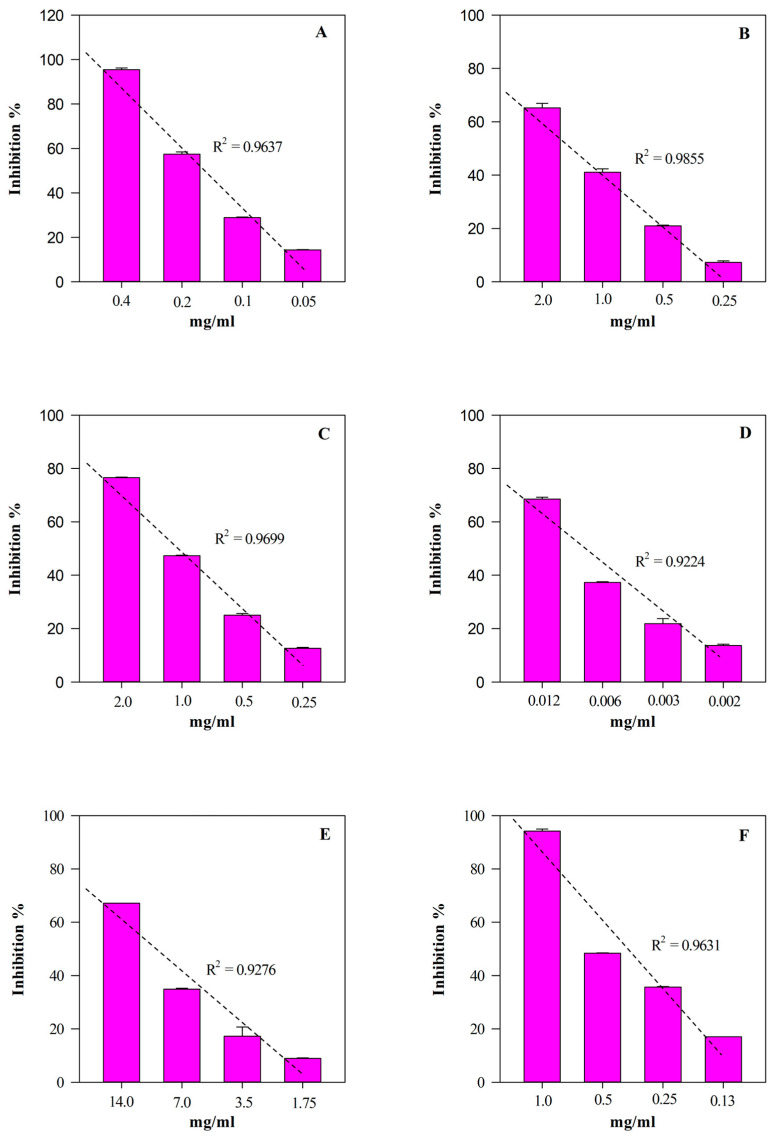
Free radical scavenging and anti-inflammatory concentration-dependent behavior of *M. didyma* flowering tops HGE evaluated by FRAP (panel **A**), DPPH (panel **B**), TEAC (panel **C**), ORAC (panel **D**), ADA (panel **E**), and PIA (panel **F**) assays. Results, expressed as inhibition percentage (%), show the mean ± standard deviation of three independent experiments in triplicate (*n* = 3).

**Figure 7:**
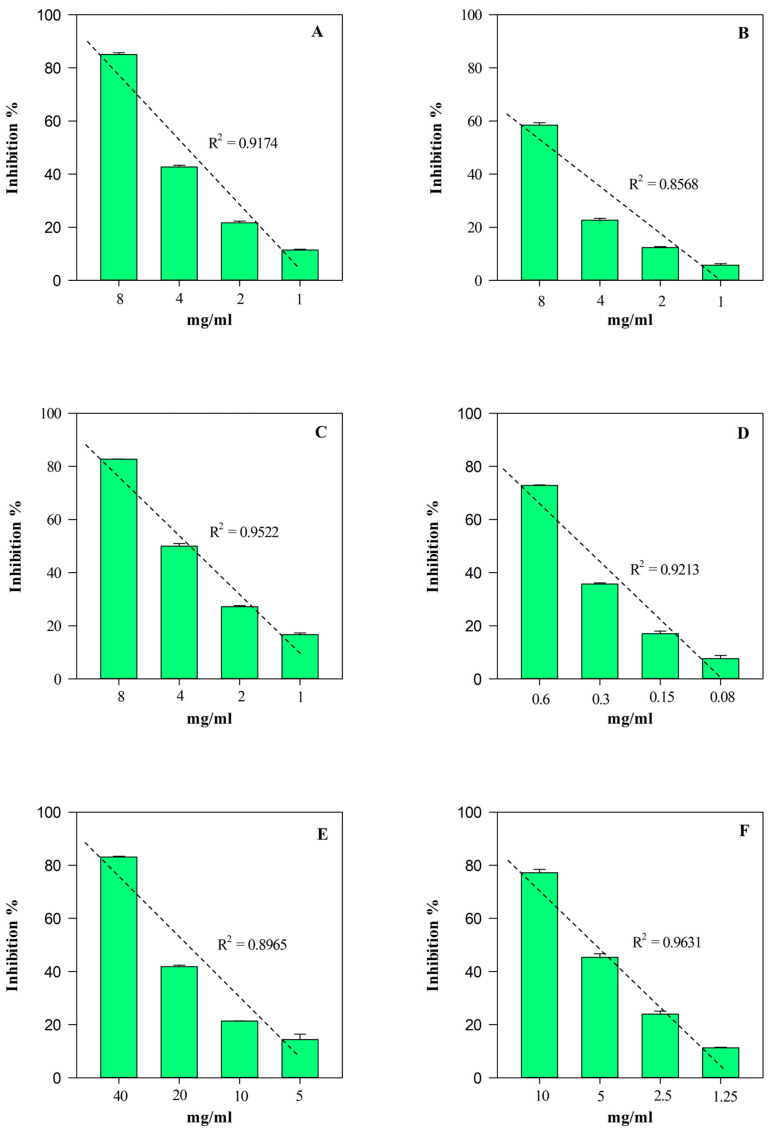
Free radical scavenging and anti-inflammatory concentration-dependent behavior of *M. didyma* flowering tops EE evaluated by FRAP (panel **A**), DPPH (panel **B**), TEAC (panel **C**), ORAC (panel **D**), ADA (panel **E**), and PIA (panel **F**) assays. Results, expressed as inhibition percentage (%), show the mean ± standard deviation of three independent experiments in triplicate (*n* = 3).

According to the phytochemical results, HGE showed the strongest and statistically significant (*p* < 0.001 vs. EE) antioxidant and anti-inflammatory activity in all the assays carried out (Table 3). The most notable biological property appears to be the antioxidant one, showing HGE IC_50_ values 5–60-times lower than those of EE. On the contrary, HGE showed an anti-inflammatory activity 2–11-times higher than EE. Furthermore, as often happens with plant extracts, both HGE and EE showed an interesting concentration-dependent antioxidant (R^2^ ≥ 0.8658) and anti-inflammatory (R^2^ ≥ 0.8965) activity, as shown in Figure 6 and Figure 7, respectively.

The strong biological properties shown by both extracts under examination, associated with the phytochemical profile detected, which is particularly rich in flavonoids, well-known vasoprotective compounds,, have led us to hypothesize a possible cosmetic use of these extracts in particular conditions such as rosacea. Considering this, to experimentally demonstrate whether these extracts were also effective in counteracting angiogenesis, typical of this pathology, a low-cost, very versatile, reproducible, and easy-to-use in vivo model, which evaluates the capacity of the extracts under investigation to inhibit angiogenesis in chicken chorioallantoic membranes (CAM assay), was used.

**Figure 8:**
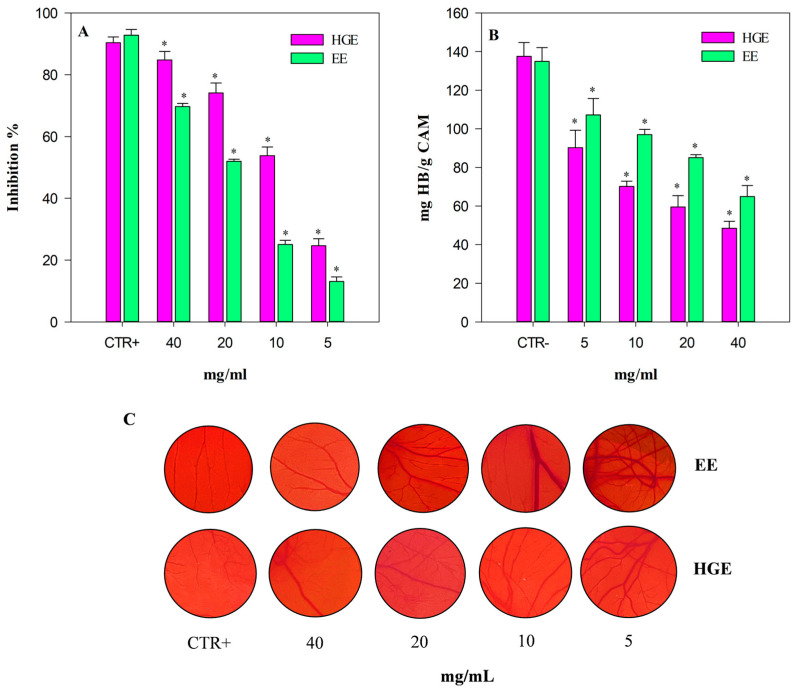
Anti-angiogenic activity of *M. didyma* flowering tops EE and HGE, investigated by CAM assay. (**A**) Antiangiogenic activity expressed as mean inhibition percentage (%) of vascular density ± standard deviation of five independent experiments in triplicate (*n* = 3). (**B**) Biochemical evaluation of the hemoglobin content. (**C**) Representative pictures of the CAM treated with CTR+ (retinoic acid 10 µg/mL), EE, and HGE at the same concentration range (5–40 mg/mL). * *p* < 0.001 vs. the respective CTR+ (purple and green bar for HGE and EE, respectively).

The angiogenesis inhibition, evaluated by a semi-quantitative analysis of the number of blood vessel branch points in a standardized area by a stereomicroscope, and expressed as inhibition percentage (%) with respect to the positive control (CTR+, 10 µg/mL retinoic acid), is shown in Figure 8, panel A. As expected, HGE (purple bars) shows once again the strongest activity, with statistically significant (*p* < 0.001) values compared to EE (green bars). Specifically, HGE showed an anti-angiogenic activity approximately double that of EE with the following IC_50_ value: 10.17 mg/mL (8.38–12.35) vs. 20.65 mg/mL (16.98–25.11), respectively. Both extracts showed statistically significant results (*p* < 0.001) with respect to the positive control retinoic acid: IC_50_ 4.52 µg/mL (3.28–5.78).

These results have been corroborated also by the biochemical determination of the hemoglobin content of each treated CAM (Figure 8, panel B).

The CAM hemoglobin (Hb) content, expressed as mg Hb/g CAM, shows a trend inversely proportional to the anti-angiogenic activity. Indeed, the hemoglobin content is significantly lower in CAM treated with HGE, with results statistically significant vs. EE (*p* < 0.001). Figure 8 panel C shows representative pictures of the analyzed CAM-standardized area of the CTR+, EE, and HGE (5–40 mg/mL).

## 3. Discussion

Exploring bioactive compounds from natural sources is an important tool for the discovery of new potential agents useful for both therapeutic and cosmetic applications. Considering this, we studied flowering tops of *M. didyma* to investigate a potential skincare application of crude food-grade extracts. Recent studies on the essential oil of this species revealed the presence of several bioactive compounds with healthy properties [14,17]. On the contrary, the extract needs further investigations to detect the type and number of phenolic compounds that could be involved in the biological activity of the plant complex, and to evaluate new potential nutraceutical and cosmeceutical applications. Additionally, a detailed study on the anatomy of *M. didyma* is lacking. Therefore, a micro-morphological characterization of the flowering tops aimed at identifying the main sites of accumulation of the secondary metabolites within plant tissues was also carried out. Like in most Lamiaceae species, several types of non-glandular and glandular trichomes, including different types of capitate and peltate trichomes, were widely distributed on the aerial parts of *M. didyma*. These features are important in the quality control of the raw herbal material because the morphology and distribution of plant trichomes are considered an important taxonomic character in this family [18,19].

The scarlet-red flowers and bracts were rich in anthocyanins, which are probably involved in the plant protection against biotic and abiotic stressors [20], and in the pollinator’s attraction [21,22]. As well as within the epidermal cells of flowers and bracts, these red pigments were also found in solution inside the cell vacuoles of the NGTs, and within the stalk cells of the long capitate ones. Similarly, Jordheim et al. [20] detected anthocyanins within most cells of non-glandular cauline hairs of *Plectranthus ciliatus* E. Mey. (Lamiaceae).

In *M. didyma*, the stalk cell of the Type I CGTs appeared reddish-pink too, due to the presence of these pigments. To the best of our knowledge, this was previously documented only in *Rubus odoratus* L. and *Dictamnus albus* L. [23]. In this kind of CGTs, the neck cells had thickened sidewalls that reacted positively to the test for suberin-like substances. Therefore, the role of the neck cell is probably to ensure a controlled circulation of substances between the secretory head and the stalk of the trichome [24]. Particularly, as previously described in other Lamiaceae [25], as well as in Droseraceae, Lentibulariaceae, and Bignoniaceae [26] species, it prevents the backflow of secreted substances through the apoplast.

The choice of the appropriate solvent or extraction mixture to recover the phytochemicals of interest represents the critical step in the sample preparation [27]. Generally, the organic solvents are the most widely used because, thanks to their strong eluotropic power, they can extract, with high yield, bioactive compounds with very different physical–chemical properties. However, most of them are potentially harmful for human health and the environment, and consequently their use in nutraceutical or cosmetic field is not allowed [27]. With the advent of green chemistry, we are looking with ever-greater interest at alternative extraction solvents, ranging from water, ethanol, hydroalcoholic mixtures [28], to other alternatives, such as glycerin [29]. Despite being little-used, glycerin offers numerous advantages such as no combustibility, low cost and wide availability, low volatility, and easy blending with water, with high extraction yields [30,31,32,33]. Indeed, it has been demonstrated that glycerin, and even more so the hydroglyceric blends, has a significantly higher eluotropic power on polyphenols than hydroalcoholic mixtures, especially when ethanol is used at percentages higher than 50% [34]. This aspect takes on even more importance in the cosmetic field, where the liquid extracts are the most widely used. From this point of view, other than being rich in bioactive compounds, it must guarantee, in addition to safety, stability, and a long shelf-life, also suitable technological properties, supporting the development of skincare formulations without affecting their physical-chemical properties and stability [35].

It has been demonstrated, according to our results, that the use of the hydroglyceric mixture increased specifically the flavonoid content of plant extracts [31,34].

These compounds have promising applications, especially in the cosmetic field, and can be used instead of synthetic compounds to obtain effective and safe natural products, in line with the current market demands [36]. The main health properties of flavonoids are related to their antioxidant, anti-inflammatory, antimicrobial, anti-angiogenic, and anti-melanogenic activity [37,38,39,40]. Considering this, they are currently used in several anti-aging and skin-lightening cosmetics [41,42], but certainly new applications could be explored.

Beyond the extractive mixture, a key role is played by the chosen plant material to recover those phytochemicals. In this regard, the Lamiaceae family represent a great source of valued plant species [43]. Among these, *Monarda* spp. are still poorly investigated, especially as regards non-volatile compounds. A recent study investigated the phytochemical profile of different methanolic extracts from flowering herbs of *Monarda* spp. [44]. Eighteen polyphenols, belonging to the phenolic acids and flavonoids classes, were identified. Among these, according to our results, authors identified hydroxybenzoic and rosmarinic acid, as well as coumaric, kaempferol, luteolin, and apigenin derivatives [44]. However, no anthocyanins were detected [44].

The presence of these compounds, according to our results, has been observed in previous studies examining *M. didyma* and *M. fistulosa* flower and leaf extracts [45,46,47,48]. Polyacylated anthocyanins, flavonols such as quercetin and apigenin glycosides, and the respective aglycons were identified both in *M. didyma* leaf and flower extracts [45,46]. Moreover, the flower extract is the richest in flavonoids, probably due to the most abundant presence of anthocyanins, according to our phytochemical and micro-morphological results. These data were also in accordance with what was observed in the *M. fistulosa* flower extract, in which several polyacetylated anthocyanins, as well as cumaric and apigenin derivatives, have been identified [47,48]. Moreover, rosmarinic acid, as well as quercetin and apigenin glycosides, was previously identified, in agreement with our results, in the *M. didyma* herb by high-performance thin-layer chromatography [49]. On the contrary, this is the first study which highlighted the presence of epicatechin derivatives, as well as lignans and other minor compounds such as rosmadial and ligstroside, in *M. didyma* flowering tops extracts. Among other things, to our knowledge, this is the first study that has qualitatively and quantitatively analyzed the phytochemical profile of *M. didyma* flowering tops.

The few studies available on *M. fistulosa* extracts demonstrate how the polyphenols identified in plants of the *Monarda* genus can play a pivotal role from a pharmacological point of view, exhibiting strong anti-inflammatory, antiradical, antinociceptive, and analgesic properties [50]. They act directly as a powerful free radical scavenger as well as reducing and anti-peroxidative agents, and, indirectly, by modulating the cellular defences via the NF-E2-related factor 2-antioxidant-responsive element pathway, which regulates the expression of several antioxidant genes [51].

These properties correlate positively with the number of free hydroxyl groups present on the phenolic structure and negatively with the glycosylation degree [52,53,54]. Among the identified polyphenols, certainly flavonols, flavan-3-ols, and anthocyanins play a pivotal role in the higher antioxidant, anti-inflammatory, and anti-angiogenic activity detected in HGE [55,56]. Flavonols, the second most abundant class of flavonoids detected in HGE, showed a promising anti-angiogenic activity due to the presence of a C2–C3 double bond conjugated with a 4-oxo function and the presence of a 3-hydroxyl group in the C ring [56]. Indeed, flavanones, which lack these characteristics, show a weaker biological activity. On the contrary, some flavan-3-ols, which possess intermediate features such as a 3-hydroxyl group in the C ring, a catechol structure, and free hydroxyls, can contribute to the observed anti-angiogenic activity [40]. Considering this, kaempferol, quercetin, and epicatechin derivatives, the most abundant compound detected in HGE belonging to the polyphenolic classes mentioned above, play a pivotal role. Indeed, it has been demonstrated that they have strong anti-angiogenic properties by inhibiting several signalling pathways such as VEGFR2, MEK/ERK, PI3K/AKT, and MEK/JNK [57]. Among anthocyanins, cyanidin 3-*O*-sambubioside and other cyanidin derivatives, characterized by the catechol structure, show a stronger activity with respect to other anthocyanins with the same hydroxylation pattern in the A and C rings but with only one OH group in the B ring, such as pelargonidin, malvidin, and peonidin [40].

Finally, other compounds, which certainly contribute to the antioxidant and anti-inflammatory pattern of HGE, are phenolic acids, whose reducing ability depends on the number of free hydroxyl groups [58]. According to this, among the phenolic acids identified in HGE, sinapoylquinic acid plays a pivotal role, followed by chlorogenic and p-hydroxybenzoic acid.

The anti-inflammatory activity of hydroxycinnamic acids is well-known thanks to several in vitro and in vivo studies by which it has been demonstrated that these compounds act predominantly by decreasing the expression of inflammatory mediators such as the interleukin-6, IL-1, and tumor necrosis factor-α (TNF-α) [59], as well as by decreasing the expression of nuclear factor kappa-light-chain-enhancer of activated B cells p65. This latter factor has recently been correlated to the angiogenesis process by regulating key angiogenesis factors such as the vascular endothelial growth factor, transforming growth factor beta, IL-6, and TNF-α [60].

Therefore, the choice to use a plant extract rich in polyphenols rather than single pure compounds lies in the fact of fully exploiting these additive or sometimes synergistic effects, which allow the simultaneous modulation of different intracellular pathways and cellular targets, with the final goal being to obtain the best biological activity.

## 4. Materials and Methods

### 4.1. Plant Material

*M. didyma* is an herbaceous perennial plant growing up to 1.5 m and blooming from midsummer to autumn. The dark-green leaves are opposite, ovate–lanceolate and acuminate, with serrated margins (7.5–15 cm long).

**Figure 9:**
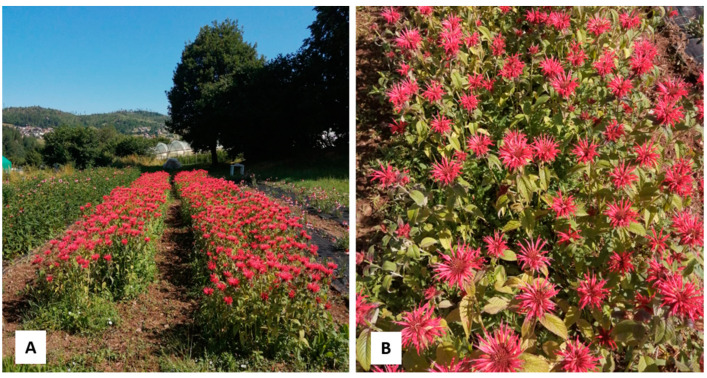
Plantation of *M. didyma* L. (**A**) and detail of the flowering tops (**B**).

The flowering tops were harvested at the end of June, during the flowering stage, from the experimental plots in Baselga di Piné, frazione Miola, Località Fovi (TN), located about 960 m a.s.l. (Figure 9). A voucher specimen (GDOR n. 60698) was deposited in the herbarium of the Natural History Museum Giacomo Doria of Genova (Italy).

The flowering tops were air-dried in the shadow at room temperature and frozen at −18 °C to avoid attack by insects, larvae, and parasites. EE and HGE were made by using the Naviglio^®^ Extractor (Atlas filtri engineering, Padua, Italy). In both cases, 200 g of dried flowering tops were placed in a filter bag with a porosity of 50 microns, placed inside the vessel of the Naviglio^®^ extractor and added, respectively, with 1.34 kg of 96% ethanol and 2.37 kg of water/glycerin solution (1:4, *v*/*v*). The drug/solvent ratio was chosen according to the relative density of the solvents used to reach the loading volume and recommended working pressure (6–7 bar at starting time) of the extractor chamber. The extraction process involved 15 cycles (7 min static phase followed by 1 min dynamic phase for each cycle) for a total run time of 2 h. The extracts were then recovered by emptying the instrument and subsequently by squeezing the starting plant material. One and twenty-eight (1.28) kg of EE (drug/extract ratio 1:6.4), and 1.82 kg of HGE (drug/extract ratio 1:9), were obtained.

### 4.2. Microscopical Analyses

Fresh flowers, red-tinged bracts, and ovate–lanceolate green leaves located at the end of the branching stems were firstly observed by stereomicroscope (LEICA M205 C, Leica Microsystems, Wetzlar, Germany) to assess their general features. Additionally, investigations of the main anatomical and micro-morphological characteristics were performed by a Leica DM 2000 transmission light microscope, equipped with a ToupCam Digital Camera and CMOS Sensor with a 3.1 MP resolution (ToupTek), and by SEM using a Vega3 Tescan LMU microscope (Tescan USA Inc., Cranberry Twp, PA, USA), operating at an accelerating voltage of 20 kV.

For optical microscope observations, leaf epidermal peels were obtained from fresh samples by directly tearing off the lower epidermis layer with a tweezer. Cross-sections of leaves, bracts, and flowers were hand-made using a double-edged razor blade. All these samples were then mounted in water and directly observed to pinpoint the anthocyanins or stained with Fluorol Yellow 088 to detect the presence of lipids in the plant tissues [61]. In addition, to better characterize leaf and flower anatomy, small specimens were cleared with an aqueous solution of chloral hydrate and mounted in a chloral hydrate–glycerol solution to prevent crystallization of the reagent during the observation of the slides, according to Jackson and Snowdon [62].

For SEM analyses, leaves were fixed in a 70% ethanol–FineFix solution (Milestone SRL, Sorisole, Bergamo, Italy) for 24 h at 4 °C. Afterwards, the samples were dehydrated in a series of solutions with increasing ethanol content [63]. Samples were then critical point dried (CPD, K850 2M Strumenti s.r.l., Rome, Italy), mounted on aluminium stubs, using conductive double-sided adhesive carbon tapes, and finally sputter-coated with a 10 nm layer of gold [64].

### 4.3. Phytochemical Analyses

#### 4.3.1. Total Phenolic Compounds

Total phenols were quantified according to Ingegneri et al. [65]. Briefly, 10 µL of EE and HGE (12.5–100 mg/mL and 5.0–40 mg/mL for EE and HGE, respectively) was added to 90 µL of deionized water and Folin–Ciocalteu reagent (100 µL). After 3 min, 10% sodium carbonate (100 µL) was added, incubating the samples in the dark at RT for 60 min, vortex-mixing every 10 min. Absorbance was read at 785 nm by using a UV–Vis reader plate (Multiskan GO; Thermo Scientific, Waltham, MA, USA) against a blank consisting of ethanol and hydroglyceric mixture, respectively. Gallic acid was used as a reference compound (0.075–0.6 mg/mL), and results were expressed as g of gallic acid equivalents (GAE)/100 g of liquid extract (LE).

#### 4.3.2. Total Flavonoids

Total flavonoids were quantified according to Lenucci et al. [66]. Briefly, 50 µL of EE and HGE (12.5–100 mg/mL and 5.0–40 mg/mL for EE and HGE, respectively) was added to 450 µL of deionized water, followed by 30 µL of 5% NaNO_2_. After 5 min incubation at RT, 60 µL of 10% AlCl_3_ was added, and samples incubated again for 6 min. Two-hundred microliters of 1 M NaOH and 210 µL of deionized water were added and vortex-mixed. The absorbance was recorded at 510 nm by using an UV–Vis spectrophotometer (Model UV-1601, Shimadzu, Kyoto, Japan).

Rutin was used as a reference compound (0.125–1.0 mg/mL), and results were expressed as g of rutin equivalents (RE)/100 g LE.

#### 4.3.3. Anthocyanins

Quantitative analysis of anthocyanins was carried out according to Rapisarda et al. [67]. Briefly, 2 mL of liquid extract (EE and HGE) was added in twice to a 25 mL flask and brought to the volume with 0.2 M KCl (pH 1 with 0.2 M HCl) and 1 M CH_3_CO_2_Na (pH 4.5 with 1 M HCl), respectively. Absorbance of the two solutions was recorded at 510 nm and the anthocyanins concentration calculated according to the following equation:

$$C_{mg/100mL}=\left({Abs}_{pH1}-{Abs}_{pH4.5}\right)\;\times \;484.82\;\times 100/24825\;\times \;DF$$

where Abs_pH1_ and Abs_pH 4.5_ are the absorbance of the pH 1 and pH 4.5 sample solutions, respectively, 482.82 is the molecular mass of cyanidin-3-*O*-glucoside chloride, 24,825 is its molar absorptivity (ε) at 510 nm in the pH 1 solution, and DF is the sample dilution factor.

#### 4.3.4. Flavan-3-ols

The quantification of flavan-3-ols was carried out by vanillin index assay, based on the ability of vanillin to react, in an acid environment, with the free carbons C6 and C8 of flavan-3-ols [68,69]. Briefly, 0.5 mL of EE and HGE was added to 1.5 mL 0.5 M sulphuric acid and loaded onto a conditioned Sep-Pak C18 cartridge (Waters, Milan, Italy), washed with 2.0 mL of 5.0 mM sulphuric acid, and eluted with 5.0 mL of methanol. One millilitre of each eluate was added to 6.0 mL of 4% vanillin methanol solution for 10 min. After cooling, 3 mL of HCl was added, and after 15 min incubation the absorbance was recorded at 500 nm using the same instrument and blank reported in Section 4.3.2 and Section 4.3.1, respectively. Catechin was used as a reference compound (0.125–0.50 mg/mL). Results were expressed as g of catechin equivalents (CE)/100 g LE.

#### 4.3.5. Proanthocyanidins

Proanthocyanidins were indirectly detected by hot-hydrolyzation in an acid environment [68,69,70]. Briefly, 400 µL of EE and HGE diluted to 2 mL with 0.05 M sulphuric acid were loaded onto a conditioned Sep-Pak C18 cartridge (Waters, Milan, Italy). The proanthocyanidin-rich fraction obtained was eluted with 3 mL methanol and collected in a 100 mL round bottom flask shielded from light and containing 9.5 mL of absolute ethanol. After this, 12.5 mL of 300 mg/L ferrous sulphate heptahydrate diluted in HCl was added. Samples were refluxed for 50 min. After cooling, the absorbance was recorded at 550 nm using the same instrument and blank reported in Section 4.3.2.

The proanthocyanidin content was expressed as five-times the amount of cyanidin formed by means of a cyanidin chloride (ε = 34,700) calibration curve, by subtracting the basal anthocyanidins content, obtained by processing the samples in the same manner but without heating. Results were expressed as g of cyanidin equivalents (CyE)/100 g LE.

#### 4.3.6. LC-DAD-ESI-MS Analysis

The phytochemical profile of EE and HGE was elucidated by LC-DAD-ESI-MS analysis. Separation was carried out by a Luna Omega PS C18 column (150 mm × 2.1 mm, 5 µm; Phenomenex, Torrance, CA, USA) at 25 °C by using 0.1% formic acid (Solvent A) and acetonitrile (Solvent B) as a mobile phase according to the elution program reported in Danna et al. [71]. Five microliters of each extract were injected, and the UV–Vis spectra of analytes were recorded in the range of 190 to 600 nm. Chromatograms were acquired at 260, 292, 330, 370, and 520 nm, to detect all polyphenol classes, whereas the ion trap (model 6320, Agilent Technologies, Santa Clara, CA, USA) was carried out in full-scan mode (90–1000 *m*/*z*) following both positive and negative electrospray ionizaton (ESI) according to Danna et al. [71]. Identification was carried out by comparing the retention times and UV–Vis and MS spectra of each analyte with those of commercially available HPLC-grade standards (see Table 2), as well as with literature data and UV–Vis and mass spectra databases. Quantification was carried out by using external calibration curves of the authentic reference standard, when commercially available, or structurally similar compounds (see Table 2 footnotes). Results were expressed as mg of each compound/100 mL of LE ± standard deviation of three independent analyses in triplicate (*n* = 3).

### 4.4. Biological Activities

The antioxidant and anti-inflammatory activity of EE and HGE were evaluated by several in vitro spectrophotometric and spectrofluorimetric assays based on different mechanisms and reaction environments, whereas the anti-angiogenic activity was evaluated by the in vivo CAM assay. The results, which represent the average of three independent experiments in triplicate (*n* = 3) for the in vitro assay and five independent experiments in triplicate (*n* = 3) for the in vivo assay, were expressed as the inhibition (%) of the oxidative/inflammatory/angiogenic activity, by calculating the IC_50_ (mg/mL) with the respective C.L. at 95% by Litchfield and Wilcoxon’s test (PHARM/PCS 4, MCS Consulting, Wynnewood, PA, USA). All concentration ranges reported below refer to the final concentrations of EE, HGE, and reference compounds within the reaction mixture.

#### 4.4.1. FRAP Assay

The FRAP assay was carried out according to Ingegneri et al. [65]. Briefly, 10 µL of EE and HGE (1.0–8.0 mg/mL and 50–400 µg/mL, respectively) was added to 200 µL of fresh, pre-warmed (37 °C) working reagent consisting of 300 mM buffer acetate (pH 3.6), 10 mM 2,4,6-Tris(2-pyridyl)-s-triazine dissolved in 40 mM hydrochloric acid, and 20 mM ferric chloride, and incubated for 4 min at RT in the dark. The absorbance was recorded at 593 nm using the same instrument and blank reported in Section 4.3.1. Trolox was used as a reference compound (1.25–10.0 µg/mL).

#### 4.4.2. DPPH Assay

The DPPH assay was carried out according to Ingegneri et al. [65]. Briefly, 3.75 µL of EE and HGE (1.0–8.0 mg/mL and 0.25–2.0 mg/mL, respectively) was added to 150 µL fresh 2.50 mg/mL DPPH methanol solution, mixed, and incubated in the dark for 20 min. The absorbance was recorded at 517 nm using the same instrument and blank reported in Section 4.3.1. Trolox was used as a reference compound (2.5–20.0 µg/mL).

#### 4.4.3. TEAC Assay

The TEAC assay was carried out according to Ingegneri et al. [65]. The radical reagent was prepared by mixing 1.7 mM ABTS with 4.3 mM potassium persulfate, and incubated in the dark for 12 hrs. Then, the radical solution was diluted to obtain an average absorbance of 0.7 at 734 nm and used within 4 h. Ten microliters of EE and HGE (1.0–8.0 mg/mL and 0.25–2.0 mg/mL, respectively) was added to the reagent (200 µL) and incubated at RT for 6 min. The decrease in absorbance was recorded at 734 nm by using the same instrument and blank reported in Section 4.3.1.

#### 4.4.4. ORAC Assay

The ORAC assay was carried out according to Bellocco et al. [54]. Briefly, 20 µL of EE and HGE (75.0–600 mg/mL and 2.0–12.0 µg/Ml, respectively) was added to 120 µL of fresh 117 nM fluorescein and incubated for 15 min at 37 °C. Then, 60 µL of 40 mM AAPH was added to trigger the reaction, which was recorded every 30 s for 90 min (λ_ex_ 485; λ_em_ 520) by a fluorescence microplate reader (FLUOstar Omega, BMG LABTECH, Ortenberg, Germany). Trolox was used as a reference compound (0.25–2.0 µg/mL).

#### 4.4.5. Albumin Denaturation Assay

The ability of EE and HGE to inhibit heat-induced albumin denaturation was evaluated according to Cornara et al. [72]. Briefly, 100 µL of 0.4% fatty acid-free bovine serum albumin solution and 20 µL of phosphate buffer saline (pH 5.3) were seeded in a 96-well plate. Then, 80 µL of EE and HGE (5.0–40.0 mg/mL and 1.75–14 mg/mL, respectively) was added, and the absorbance was immediately recorded at 595 nm by using the same instrument and blank reported in Section 4.3.1. Samples were then incubated for 30 min at 70 °C, and the absorbance recorded again. Diclofenac sodium was used as a reference compound (3.0–24.0 µg/mL).

#### 4.4.6. Protease Inhibition Assay

The protease inhibitory activity was evaluated according to Cornara et al. [72]. Briefly, 200 µL of EE and HGE (1.25–10 mg/mL 0.125–1.0 mg/mL, respectively) was added to a reaction mixture consisting of 12 µL trypsin (10 µg/mL) and 188 µL Tris-HCl buffer (25 mM, pH 7.5). Two-hundred microliters of 0.8% casein was added and the reaction mixture was incubated for 20 min at 37 °C in a water bath. The reaction was stopped by adding 400 µL of perchloric acid. The cloudy suspension was centrifuged at 3500× *g* for 10 min, and the absorbance of the supernatant was recorded at 280 nm using the same instrument and blank reported in Section 4.3.1. Diclofenac sodium was used as the reference standard (5.0–40.0 µg/mL).

#### 4.4.7. CAM Assay

Anti-angiogenic effects were evaluated using the CAM assay according to Smeriglio et al. [73]. Briefly, fertilized eggs of *Gallus gallus* were incubated for 4 days at 37 °C. After this, an incision (1 cm^2^) was made on the apical part of the egg, to remove the shell and visualize the CAM. Eggs with malformed or dead embryos were discarded. EE and HGE solutions (100 µL), properly diluted in albumen (5.0–40.0 mg/mL), were applied directly on the CAM surfaces. Retinoic acid (10 µg/mL) was used as a positive control (CTR+). CAMs treated only with the sample vehicle (ethanolic and hydroglyceric solution) were also included as negative controls (CTR-). After treatment, the eggs were incubated for 48 h, monitoring the CAM development. At the end of the experiment, the number of blood vessel branch points was evaluated in a standardized area by a stereomicroscope (SMZ-171 Series, Motic). Pictures were acquired by a digital camera (Moticam^®^ 5 plus) and analyzed by the GNU Image Manipulation Program (GIMP version 2.10.2). Results were expressed as reported in Section 4.4.

The angiogenesis inhibition was also assessed biochemically, according to Certo et al. [74] with some modification, by determining the Hb content of each CAM. CAM tissues (1 g) were homogenized (IKA ULTRATURRAX^®^ T45, IKA^®^-Werke GmbH & Co. KG, Staufen, Germany) for 1 min with 1.15% KCl (2 mL). After centrifugation at 3000× *g* 15 min at RT, the supernatant (20 µL) was mixed with fresh Drabkin’s reagent (5 mL) and incubated for 15 min in the dark at RT. The absorbance was recorded at 540 nm using the same instrument reported in Section 4.3.2. Results were expressed as mg of Hb/g of CAM, using an external standard Hg calibration curve (45–720 µg/mL).

### 4.5. Statistical Analysis

The statistical significance was evaluated by a one-way analysis of variance (ANOVA), followed by a Student–Newman–Keuls and Tukey’s test using SigmaPlot 12.0 software (Systat Software Inc., San Jose, CA, USA). *p* < 0.05 was considered statistically significant.

Moreover, agglomerative two-way hierarchical clustering analysis was carried out to highlight the phytochemical relationships among the two extracts investigated using the statistical JMP7 for SAS software (version 7, SAS Institute Inc., Cary, NC, USA). Euclidean distances were used to measure the dissimilarity between samples, whereas hierarchical clustering was performed according to Ward’s minimum variance method.

## 5. Conclusions

*M. didyma* is a very interesting plant because it is rich in secondary metabolites with strong healthy properties. The presence of the flowering tops of this plant in the BELFRIT list finds its rationale, as demonstrated by the present study in which 53 polyphenols were identified and quantified, some of them for the first time. Indeed, the use of flowering tops rather than just leaves or flowers offers a much wider range of bioactive compounds than has been highlighted to date. Beyond flavonols and flavan-3-ols, which represent the predominant compounds, it is also an important source of other classes of flavonoids, including anthocyanins, and mono- and poly-glycosylated phenolic acids.

The choice of the appropriate extraction solvent in the preparation of an extract for nutraceutical and cosmeceutical use is important, and this study, which compares two different food-grade extracts, demonstrates that although the two extracts show a common phytochemical profile, the extraction solvent influences, sometimes even conspicuously, the content of bioactive compounds and consequently the biological activity observed.

The hydroglyceric extract proved to be the most promising in all tests carried out both in vitro and in vivo. Certainly, the biological tests carried out in this first study are still preliminary and require further investigation with more complex cellular models, but they can already drive towards the choice of a suitable extraction technique to obtain a liquid extract with potential skincare applications.

## Figures and Tables

**Table 1:** Phytochemical screening of *M. didyma* flowering tops EE and HGE. Results are the mean ± standard deviation (S.D.) of three independent experiments in triplicate (*n* = 3).

Phytochemical Assay	EE	HGE
Total phenols (mg GAE ^a^/100 mL LE ^b^)	64.22 ± 3.45	105.75 ± 5.91 *
Flavonoids (mg RE ^c^/100 mL LE)	47.70 ± 1.27	71.60 ± 5.09 *
Anthocyanins (mg CyGE ^d^/100 mL LE)	0.35 ± 0.02	3.22 ± 0.15 *
Vanillin index (mg CE ^e^/100 mL LE)	5.83 ± 0.22	43.89 ± 1.88 *
Proanthocyanidins (mg CyE ^f^/100 mL LE)	0.004 ± 0.00	0.022 ± 0.00 *
Polimerization index ^g^	1623.73	1971.60 *

^a^ GAE, Gallic acid equivalents; ^b^ LE, Liquid extract; ^c^ RE, Rutin equivalents; ^d^ CyGE, Cyanidin-3-*O*-glucoside equivalents; ^e^ CE, Catechin equivalents; ^f^ CyE, Cyanidin equivalents; ^g^ Polymerization index = vanillin index/proanthocyanidins, both expressed as mg/100 mL LE. * *p* < 0.05 vs. EE.

**Table 2:** Qualitative and quantitative analysis of phytochemicals within the ethanolic and hydrogliceric extracts (EE and HGE, respectively) of *M. didyma* flowering tops by LC-DAD-ESI-MS.

Compound	RT ^a^ min	λ_max_ nm	[M-H]^−^ m/z	[M-H]^+^ m/z	EE	HGE
					mg/100 mL LE ^b^	
Quinic acid ^c^	1.6	233; 310	191	-	0.87 ± 0.03	1.88 ± 0.05 *
Sinapoylquinic acid ^d^	6.4	218, 327	397	-	0.17 ± 0.01	0.07 ± 0.00 *
(-)-Epigallocatechin 3-*O*-gallate ^c^	10.1	274	457	-	2.29 ± 0.06	14.66 ± 0.85 *
1-*O*-Feruloyl-beta-D-glucose ^c^	13.8	330	355	-	0.22 ± 0.01	0.19 ± 0.01
Rosmadial ^e^	15.3	234, 290, 356	343	-	1.63 ± 0.03	2.48 ± 0.08 *
Anhydro-secoisolariciresinol ^f^	16.6	288	343	-	0.18 ± 0.01	0.15 ± 0.01
Peonidin 3-*O*-(6″-acetyl-glicoside) ^g^	17.3	277, 526	504	-	0.01 ± 0.00	0.01 ± 0.00
Pelargonidin 3,5-di-beta-D-glucoside (Monardin) ^c^	19.8	276, 329, 501	-	595	0.01 ± 0.00	0.06 ± 0.00 *
3-*O*-Caffeoyl quinic acid (Chlorogenic acid) ^c^	20.3	248, 326	353	-	1.83 ± 0.03	2.70 ± 0.06 *
4-Hydroxybenzoic acid ^c^	22.4	253	137	-	6.89 ± 0.22	8.02 ± 0.17 *
(+)-Catechin 3-*O*-glucoside ^h^	22.8	276, 320	-	453	1.71 ± 0.04	15.61 ± 0.76 *
Bis (demalonyl) monardaein (Monardein) ^i^	24.0	286, 313, 507	-	742	0.01 ± 0.00	0.03 ± 0.00 *
Apigenin-7-*O*-glucuronopyranosyl(1,2)-glucuronopyranoside ^j^	25.2	267, 336	-	622	0.08 ± 0.00	0.13 ± 0.00 *
(-)-Epigallocatechin 3-*O*-glucuronide ^k^	27.3	272	-	483	0.25 ± 0.01	4.28 ± 0.12 *
Kaempferol 3-*O*-(6″-malonyl-glucoside) ^l^	28.6	265, 345	533	-	9.65 ± 0.24	8.53 ± 0.18 *
Ligstroside ^c^	29.1	235, 275	-	525	0.06 ± 0.00	0.07 ± 0.00
Delphinidin 3-*O*-sambubioside ^c^	30.2	274, 523	596	-	0.01 ± 0.00	0.17 ± 0.01 *
Diosmin ^c^	30.4	260,350	-	609	0.36 ± 0.02	0.45 ± 0.02 *
Phloretin 2′-*O*-xylosyl-glucoside ^m^	30.8	242, 289	-	569	0.83 ± 0.04	0.65 ± 0.03 *
Malvidin 3-*O*-(6″-p-coumaroyl-glucoside) ^n^	31.2	274, 310, 527	638	-	0.01 ± 0.00	0.02 ± 0.00
Chicoric acid ^c^	31.7	250, 330	-	475	0.08 ± 0.00	0.07 ± 0.00
Kaempferol 3-*O*-xylosyl-glucoside ^l^	32.4	253, 266, 323, 364	579	-	2.73 ± 0.08	1.71 ± 0.02 *
Kaempferol 3-*O*-rhamnosyl-rhamnosyl-glucoside ^l^	33.6	253, 265, 325, 364	-	741	0.75 ± 0.03	0.55 ± 0.02 *
Cyanidin 3,5-*O*-diglucoside ^c^	33.7	279, 326, 514	610	-	0.03 ± 0.00	0.01 ± 0.00 *
Quercetin 3-*O*-xylosyl-rutinoside ^o^	34.0	258, 360	-	743	2.55 ± 0.07	1.55 ± 0.10 *
Rosmarinic acid ^c^	34.4	292, 332	359	-	0.02 ± 0.00	0.03 ± 0.00
Delphinidin 3-*O*-glucoside ^c^	35.5	274, 523	464	-	0.01 ± 0.00	0.02 ± 0.00
Kaempferol 3-*O*-glucuronide ^p^	36.2	272, 368	461	-	0.26 ± 0.01	0.19 ± 0.01 *
Vitisin A ^n^	36.6	256, 515	-	562	0.05 ± 0.00	0.07 ± 0.00
Malvidin 3-*O*-glucoside ^c^	36.9	274, 527	-	494	0.01 ± 0.00	0.02 ± 0.00
Kaempferol-3-*O*-rutinoside ^c^	39.2	266, 348	593	-	4.23 ± 0.21	1.91 ± 0.08 *
Cyanidin 3-*O*-sambubioside ^c^	39.6	280, 517	-	617	0.10 ± 0.01	2.22 ± 0.14 *
Cyanidin 3-*O*-(6″-dioxalyl-glucoside) ^q^	41.0	280, 526	-	594	0.02 ± 0.00	0.22 ± 0.01 *
Luteolin 7-*O*-diglucuronide ^r^	42.0	253, 267, 292, 348	-	639	0.01 ± 0.00	0.02 ± 0.00
Cyanidin 3-*O*-(3″,6″-*O*-dimalonyl-glucoside) ^q^	43.9	280, 522	-	622	0.01 ± 0.00	0.11 ± 0.01 *
3-Hydroxyphloretin 2′-*O*-xylosyl-glucoside ^q^	45.8	242, 289	583	585	3.46 ± 0.12	3.95 ± 0.20 *
Secoisolariciresinol ^c^	46.2	229, 281	-	363	0.02 ± 0.00	0.03 ± 0.00
Quercetin 3′-sulfate ^s^	47.3	255, 270, 303, 370	-	383	0.43 ±0.01	0.10 ± 0.00 *
Phloretin ^c^	47.5	242, 289	-	275	0.16 ± 0.01	0.20 ± 0.01
Kaempferol ^c^	50.2	266, 366	285	-	4.92 ± 0.22	2.35 ± 0.08 *
Procyanidin dimer B type ^h^	54.1	233, 279	577	-	0.01 ± 0.00	0.03 ± 0.00 *
Luteolin 7-*O*-(2-apiosyl-6-malonyl)-glucoside ^t^	61.8	227, 348	-	667	0.12 ± 0.01	0.19 ± 0.01 *
Kaempferol 3-*O*-(6″-acetyl-galactoside) 7-*O*-rhamnoside ^l^	64.2	245, 265, 315, 350	-	637	2.25 ± 0.15	0.95 ± 0.04 *
Vanillic acid 4-sulfate ^u^	64.4	259, 292	247	-	0.03 ± 0.00	0.01 ± 0.00 *
Quercetin-3-*O*-α-L-rhamnosyl (1,2)-β-D-glucoside-7-*O*-α-L-rhamnoside ^o^	65.3	256, 374	-	757	2.48 ± 0.17	0.75 ± 0.04 *
Epigallocatechin 3-*O*-gallate-7-*O*-glucoside-4″-*O*-glucuronide ^v^	66.3	272	-	797	0.34 ± 0.02	5.00 ± 0.28 *
Cyanidin 3-*O*-diglucoside-5-*O*-glucoside ^w^	67.6	280, 522	-	774	0.03 ± 0.00	0.01 ± 0.00 *
Delphinidin 3-*O*-glucoside ^c^	69	276, 344, 525	-	466	0.03 ± 0.00	0.04 ± 0.00
Lariciresinol-sesquilignan ^x^	73.4	230, 280	-	557	0.15 ± 0.01	0.08 ± 0.00 *
Malvidin 3,5-*O*-diglucoside ^c^	75.2	273, 537	-	657	0.01 ± 0.00	0.01 ± 0.00
Methyl 4,6-di-*O*-galloyl-beta-D-glucopyranoside ^y^	77.7	280, 369	-	499	0.21 ± 0.01	0.12 ± 0.01 *
Kaempferol 3-*O*-glucosyl-rhamnosyl-glucoside ^l^	78.4	265, 294, 342	-	757	2.04 ± 0.08	0.54 ± 0.02 *
Lariciresinol ^c^	82.0	230, 280	359	-	0.35 ± 0.02	0.13 ± 0.01 *

^a^ RT, Retention time; ^b^ data are the mean ± standard deviation of three independent analyses in triplicate (*n* = 3), expressed as mg/100 mL liquid extract (LE). Quantification was carried out by building external calibration curves with commercially available reference standards (^c^), purchased from Merck (Darmstadt, Germany) and Extrasynthese (Geney, France); on the contrary, the other superscript letters indicate that the quantification was carried out based on the calibration curves of the following structural analogues: quinic acid (^d^), rosmarinic acid (^e^), secoisolariciresinol (^f^), peonidin-3-*O*-glucoside (^g^), catechin (^h^), monardin (^i^), apigenin-7-glucuronide (^j^), epigallocatechin (^k^), kaempferol 3-*O*-glucoside (^l^), phloridzin (^m^), malvidin-3-*O*-glucoside (^n^), quercetin-3-*O*-glucoside (^o^), kaempferol (^p^), cyanidin 3-*O*-glucoside (^q^), luteolin (^r^), quercetin (^s^), luteolin-7-*O*-glucoside (^t^), vanillic acid (^u^), (-)-epigallocatechin 3-*O*-gallate (^v^), cyanidin 3,5-*O*-diglucoside (^w^), lariciresinol (^x^), and gallic acid (^y^), respectively. * *p* < 0.05 vs. EE.

**Table 3:** Antioxidant and anti-inflammatory activity of *M. didyma* flowering tops EE and HGE in comparison with the reference standards. Results, which represent the mean ± standard deviation of three independent experiments in triplicate (*n* = 3), are expressed as the concentration inhibiting 50% of the oxidant/inflammatory activity (IC_50_) with 95% confidence limits (between brackets).

Test	EE (mg/mL)	HGE (mg/mL)	RS ^a^ (µg/mL)
FRAP	5.15 (3.65–7.52) ^b^	0.19 (0.13–0.26) ^b,c^	4.86 (3.23–5.65)
DPPH	9.92 (7.65–12.52) ^b^	1.31 (0.85–1.52) ^b,c^	13.57 (12.22–14.88)
TEAC	4.38 (2.21–6.31) ^b^	0.91 (0.52–1.31) ^b,c^	5.83 (4.12–6.55)
ORAC	0.42 (0.17–0.65) ^b^	0.007 (0.005–0.009) ^b,c^	0.83 (0.62–1.05)
ADA	21.80 (17.48–26.33) ^b^	10.23 (7.48–13.89) ^b,c^	46.42 (38.55–51.44)
PIA	4.97 (3.07–6.85) ^b^	0.47 (0.27–0.68) ^b,c^	39.87 (32.68–44.82)

^a^ RF, Reference standard: trolox for antioxidant assays (FRAP, DPPH, TEAC, and ORAC) and diclofenac sodium for anti-inflammatory assays (ADA and PIA); ^b^ *p* < 0.001 vs. RS; ^c^ *p* < 0.001 vs. EE.

## Data Availability

The data presented in this study are available in this published paper.
